# HER2 Analysis in Sporadic Thyroid Cancer of Follicular Cell Origin

**DOI:** 10.3390/ijms17122040

**Published:** 2016-12-06

**Authors:** Rosaria M. Ruggeri, Alfredo Campennì, Giuseppe Giuffrè, Luca Giovanella, Massimiliano Siracusa, Angela Simone, Giovanni Branca, Rosa Scarfì, Francesco Trimarchi, Antonio Ieni, Giovanni Tuccari

**Affiliations:** 1 Department of Clinical and Experimental Medicine, Unit of Endocrinology, University of Messina, AOU Policlinico G. Martino, 98125 Messina, Italy; Francesco.Trimarchi@unime.it; 2 Department of Biomedical Sciences and Morphological and Functional Images, Unit of Nuclear Medicine, University of Messina, AOU Policlinico G. Martino, 98125 Messina, Italy; acampenni@unime.it (A.C.); m.siracusadr@alice.it (M.S.); 3 Department of Human Pathology in Adult and Developmental Age “Gaetano Barresi”, Unit of Pathological Anatomy, University of Messina, AOU Policlinico G. Martino, 98125 Messina, Italy; giuffre@unime.it (G.G.); asimone@unime.it (A.S.); giobranca81@gmail.com (G.B.); rscarfi@unime.it (R.S.); aieni@unime.it (A.I.); tuccari@unime.it (G.T.); 4 Department of Nuclear Medicine, Thyroid and PET/CT Center, Oncology Institute of Southern Switzerland, 6500 Bellinzona, Switzerland; luca.giovanella@eoc.ch

**Keywords:** sporadic differentiated thyroid cancer, HER2 (Human Epidermal Growth Factor Receptor 2), immunohistochemistry, FISH (fluorescence in situ hybridization)

## Abstract

The Epidermal Growth Factor Receoptor (EGFR) family member human epidermal growth factor receptor 2 (HER2) is overexpressed in many human epithelial malignancies, representing a molecular target for specific anti-neoplastic drugs. Few data are available on HER2 status in differentiated thyroid cancer (DTC). The present study was aimed to investigate HER2 status in sporadic cancers of follicular cell origin to better clarify the role of this receptor in the stratification of thyroid cancer. By immunohistochemistry and fluorescence in-situ hybridization, HER2 expression was investigated in formalin-fixed paraffin-embedded surgical specimens from 90 DTC patients, 45 follicular (FTC) and 45 papillary (PTC) histotypes. No HER2 immunostaining was recorded in background thyroid tissue. By contrast, overall HER2 overexpression was found in 20/45 (44%) FTC and 8/45 (18%) PTC, with a significant difference between the two histotypes (*p* = 0.046). Five of the six patients who developed metastatic disease during a median nine-year follow-up had a HER2-positive tumor. Therefore, we suggest that HER2 expression may represent an additional aid to identify a subset of patients who are characterized by a worse prognosis and are potentially eligible for targeted therapy.

## 1. Introduction

The human epidermal growth factor receptor 2 (HER2) is a cell surface receptor belonging to the Epidermal growth factor receptor (EGFR) family of receptors, which includes four distinct, but closely related tyrosine kinase receptors: EGFR, HER2 (HER2/c-neu), HER3, and HER4 [1,2]. HER2 has no known cognate ligand and may become active upon hetero-dimerization with other family members, such as EGFR. Upon activation, EGFR and HER2 undergo dimerization and tyrosine auto-phosphorylation, thus leading to activation of proliferative and anti-apoptotic pathways, principally the MAPK, Akt, and JNK pathways. Through such effects on cell-cycle progression, apoptosis, angiogenesis, and tumor-cell motility, HER2 is implicated in the development and progression of cancer [1,2].

The HER2 gene is frequently amplified and the protein overexpressed in several human epithelial malignancies, including breast, gastric, ovarian, and colon-rectal cancers [3,4,5,6,7,8,9]. In such tumors, HER2 amplification/overexpression has been linked to a poor overall outcome and a poorly differentiated phenotype [3,4,5,6,7]. It has also been considered a useful indicator of response to specifically targeted therapies, such as trastuzumab, that inhibit the extracellular domain of HER2 [8,9]. Many studies have been addressed to determine the HER2-positive rate, mainly in breast and gastric carcinomas, utilizing a well codified scoring system [10,11], and HER2 status assessment is currently being used in such cancers to determine patient eligibility for treatment with trastuzumab [8,9,12,13]. 

Studies on HER2 have also been performed in thyroid cancer cells and tissues [14,15,16,17,18,19,20,21,22,23,24,25,26]. Interestingly, a wide variation in HER2 overexpression was reported in such studies, with positivity rates varying from 0% up to 70%, which may largely be attributed to inter-study technical and interpretive variations. Due to these conflicting findings, there is no consensus in the currently available literature regarding the potential prognostic and therapeutic value of this marker in thyroid cancer [16,18,23,24]. More recently, HER2 expression has been linked to the expression of estrogen receptors in thyroid tumor tissue [27] and associated with BRAF^V600E^ mutation and a more aggressive phenotype in familial papillary thyroid cancers (PTCs) [28]. 

In the present study, we investigate HER2 expression status in a surgical series of sporadic differentiated thyroid carcinomas of follicular cell origin to better clarify the role of this receptor in the stratification of thyroid cancer. 

## 2. Results

### 2.1. Clinical-Pathological Findings

The clinical-pathological features of the 90 differentiated thyroid cancer (DTC) patients (73 F and 17 M, mean age 51.6 ± 12.7 years, median 49 years) are summarized in Table 1. The 45 patients with PTC comprised 34 females and 11 males who ranged in ages from 28 to 71 years (median age, 49 years). Histologically, the papillary carcinomas were as follows: 16 classic variant, 21 follicular variant, 4 Hürthle cell variant, and 4 sclerosing variant. The 45 patients with follicular thyroid cancer (FTC) comprised 39 females and 6 males aged 22–76 years (median age, 55 years). The follicular carcinomas included 34 that were minimally invasive and 11 that were widely invasive. 

All patients had undergone total or subtotal thyroidectomy. In 61% of the patients, the tumor was <2 cm, pT1 according to TNM classification [29], and 11% had lymph node metastases (Table 1). No patient exhibited distant metastases at the time of surgery. 

All patients were followed up for at least five years after thyroidectomy at our Endocrine Unit (median follow-up duration 8.7 years, range 5–20 years). During follow-up, 6 out of 90 (6.7%) patients (all females, aged 43–76 years, median 45 years) developed metastases: one was affected by PTC in the classic variant pT1b stage, two by PTC follicular variant in the pT2 stage, and three by FTC in the pT3 stage. All PTC metastatic patients except for one (with lung metastases) had lymph-nodes metastases, located in the right lateral neck (*n* = 1), left lateral neck (*n* = 1), anterior central compartment (*n* = 3), and upper mediastinum (*n* = 1). The three FTC patients had lung and skeletal metastases.

### 2.2. Immunohistochemical (IHC) and Fluorescence In Situ Hybridization (FISH) Results

Twenty-seven specimens (17 PTC and 10 FTC) presented unamplified HER2 status and were therefore scored 0. The remaining 63 cases (28 PTC and 35 FTC) stained for HER2 with a variable intensity ranging from 1+ to 3+ (Table 2). A not negligible number of tumors, mostly papillary histotype (15 cases, 13 PTC and 2 FTC), exhibited a low (1+) and patchy expression of HER2, with a granular or diffuse cytoplasmic distribution of the staining. Such cases with no membranous staining were considered negative. 

**Figure 1:**
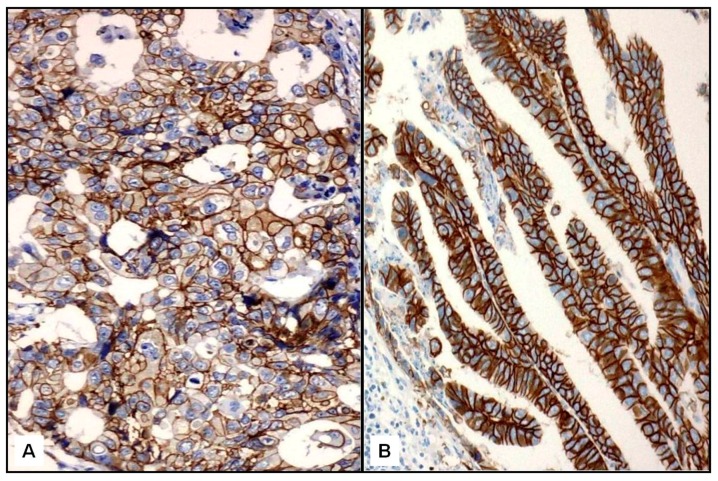
An evident diffuse membranous HER2 immunopositivity was seen in FTC (**A**, ×400) as well as in the classic variant of papillary thyroid cancer (PTC) (**B**, ×400) (Mayer’s hemalum counterstain).

**Figure 2:**
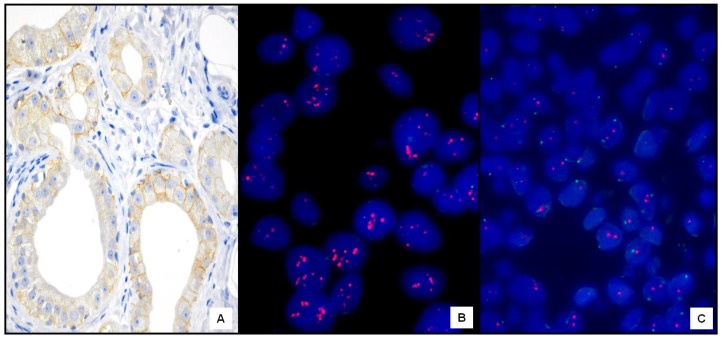
IHC equivocal (2+) follicular thyroid cancer (FTC) case (**A**, ×400) (Mayer’s hemalum counterstain) that showed a corresponding HER2 amplification by FISH (**B**, ×660). Another unamplified HER2 FTC case (**C**, ×460).

**Figure 3:**
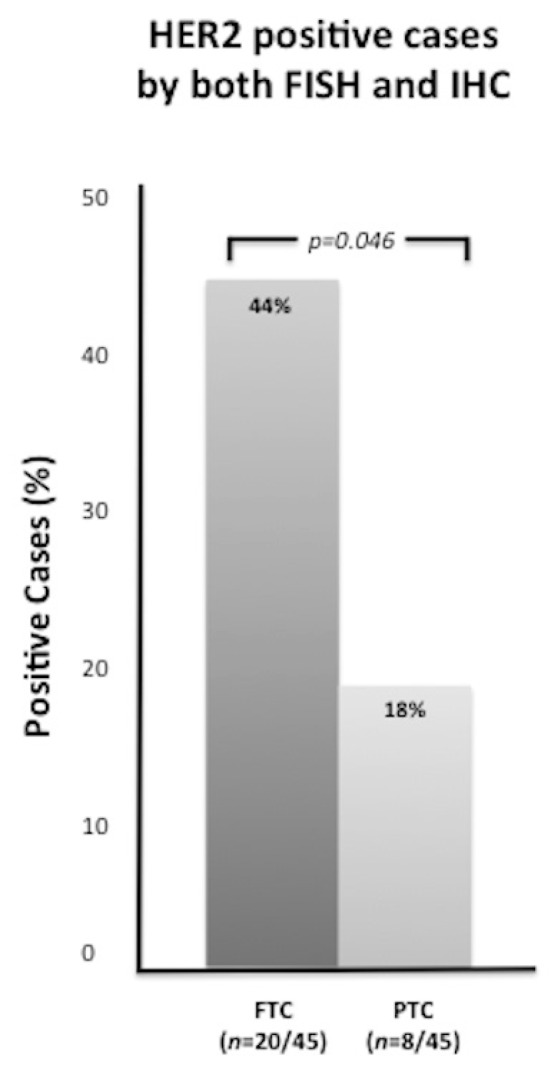
Percentages of FTC and PTC cases which tested positive for HER2 at both FISH and IHC (3+).

HER2 was clearly overexpressed (3+) at IHC with membranous staining in 26 cases: 18 FTC, of which 6 were widely invasive (see example in Figure 1A), and 8 PTC (2 classic, 5 follicular variant, and 1 Hürthle cell variant) (see example in Figure 1B). Twenty-two tumors (15 FTC and 7 PTC) were scored as 2+ by IHC for HER2. All 2+ cases (Figure 2A) were evaluated by FISH: two FTC revealed HER2 amplification (Figure 2B), while the others were unamplified (Figure 2C). Therefore, the overall rate of HER2-positive cases was 31% (28/90 cases). Specifically, HER2 amplification/overexpression was found in 20/45 (44%) FTC and 8/45 (18%) PTC, with a significant difference between the two histotypes (*x*^2^ = 3.96; *p* = 0.046) (Figure 3). Normal thyroid parenchyma surrounding the tumor lacked expression of HER2.

No significant correlation was found between HER2 expression and tumor size, as well as lymph node metastases at the time of surgery. However, among the six patients who developed metastatic disease during follow-up, five had a HER2-positive tumor. Three tumors (2 PTC and 1 FTC) stained 3+ at IHC; and the remaining two FTCs stained 2+ at IHC and revealed HER2 amplification at FISH. Thus, 5 out of 28 HER2-positive tumors and 1 out of the remaining 62 HER2-negative tumors developed metastatic disease during the follow-up (*x*^2^ = 8.18; *p* = 0.004). 

None of our cancers was iodine refractory. Concerning iodine uptake, in our cohort of DTC patients, the intensity of 131-radioiodine uptake observed (visual analysis) in thyroid remnant and, mainly, in lymph-node metastases at post-therapy whole body scan (pT-WBS) did not differ in cases overexpressing HER2 compared to negative ones.

## 3. Discussion

In the present study, we assessed HER2 status in a series of sporadic differentiated thyroid cancers (DTCs) and found that HER2 was overall overexpressed in about one-third of cases; in particular, the expression rate was significantly higher in the follicular (FTC) histotype compared to the papillary (PTC) one. 

To date, many studies have evaluated HER2 expression in thyroid cancer with controversial results, largely due to inter-study differences in the size and setting of the examined series and, most of all, to the subjective assessment and lack of uniform methodology [14,15,16,17,18,19,20,21,22,23,24,25,26]. Indeed, studies reported in the literature in the past several decades markedly differ by the methodological approach used to assess HER2 status [14,15,16,17,18,19,20,21,22,23,24,25,26]. Moreover, the criteria for scoring HER2 expression were different, and, in many studies, cytoplasmic staining patterns were reported as positive immunostaining for HER2 [16,17,18,22,23,26]. As a consequence, the results of these studies are not comparable and therefore not conclusive. 

The present study aimed to evaluate HER2 status using a method as reproducible and as standardized as possible, similar to other cancers. Indeed, a similarly wide variation in HER2 overexpression has been reported in many other tumor types [3,4,5,6,7,8]. Therefore, as the prognostic and therapeutic relevance of HER2 status has grown, the need to achieve a standardized HER2 assessment method has also arisen in neoplastic sites different from the stomach and breast [11]. In the present study, we utilized the updated ASCO-CAP scoring system reported in breast cancer [10]. Applying such strict criteria to our DTC series, we reported an overall HER2 expression of 31%. In detail, both PTC (18%) and FTC (44%) cases were found to overexpress HER2 in our series, and the expression rate was significantly different between the two histotypes in favor of the follicular one. These data may suggest a prognostic impact of HER2 status in DTC, further supported by the finding that, in nearly all patients who had metastases diagnosed during follow-up, HER2 was overexpressed in the primary tumor. Obviously, such results should be confirmed in a larger series to better determine whether HER2 amplification/overexpression can be considered an additional prognostic aid to identify cases characterized by a more aggressive disease, such as in other epithelial cancers [3,8,9,10,13].

Other studies have shown the absence of HER2 amplification in normal thyroid tissue, as well as increased expression during malignant progression in DTC [18,19,21,22]. These findings are in agreement with ours, although the importance of a standardized scoring methodology should be emphasized in order to explain the partially contradictory results on DTC. In detail, Sugishita et al. [24] investigated HER2 expression in a surgical series of 69 DTC, including 61 PTC and only 8 FTC, and found that 14 PTC and 2 FTC had a score 3+ at IHC. Amplification of HER2 gene was confirmed via FISH in 10 PTC 3+ plus 4 PTC cases 2+, and in one FTC, with an overall expression rate of 21.7% (23% considering the sole PTC). Therefore, the highest rate of HER2 expression was recorded in PTC. However, there are some limitations in the above-mentioned study [24]. First of all, the authors set an arbitrary cut-off value for the FISH ratio of 1.3 to score their cases because of the low rate of HER2 gene amplification they had found. If they had applied the HER2 amplification criteria for breast and gastric cancer (i.e., FISH ratio > 2.0) strictly to thyroid cancer, as we did, all cases would be judged as negative. Thus, these data are not comparable to ours. Secondly, the series from Sugishita et al. included only 8 FTC, without any morphological evidence of HER2 expression [24]. Successively, another surgical series of 69 DTC has been investigated for HER2 expression [25], but again a low number of FTC was included (11 cases). Although the authors utilized a HER2 scoring methodology equivalent to ours, they reported a very low rate of HER2 overexpression, since no FTC and only four (6.9%) PTCs showed HER-2 overexpression [25]. Therefore, the rate of HER2 expression in PTC was definitely lower than that previously reported elsewhere [24]; in addition, Mdah et al. [25] failed to find HER2 expression in FTC. More recently, Caria and co-workers reported a scattered HER2 expression, restricted to less than 10% of tumor cells, in a few cases of familial PTC [28]. In particular, 5/13 (38.5%) of familial PTC cases showed 5.1%–10% HER2^+^ cells, while no sporadic PTC cases exceeded the cut-off value [28]. Moreover, when familial PTCs were analyzed via IHC using an anti-c-erbB2 antibody to detect HER2 protein expression, inconsistent results compared to the FISH analysis were obtained, also possibly biased by the age of the available histological sections (7–20 years) [28]. Finally, no data about HER2 expression were recorded about different varieties present in PTC, nor in relation to the metastatic event in PTC [28].

In the present study, we analyzed a large sporadic DTC registry including the same number of PTC and FTC cases. Consequently, our data appear to be statistically more significant in comparison to the reported studies [24,25,28] and reveal a significant HER2 expression related to histotype, greatly favoring FTC. Moreover, its overexpression is more evident in metastatic DTC compared to non-metastatic ones. These findings might have potential practical implications. HER2 may be helpful in identifying a subset of DTC patients characterized by a worse prognosis, but eligible for potential targeted therapies with HER2 inhibitors, such as trastuzumab. This may be relevant in iodine-refractory cancers, since novel molecular targets and therapeutic strategies are currently under investigation for these tumors, whose treatment is still a major challenge [30,31]. Moreover, HER2 expression may be used for prognostic application in the context of other well-accepted clinic-pathological prognostic parameters for DTC (age, gender, pTNM stage, histological subtype), since very few new markers revealed prognostic value *per se* [32,33]. If our observations are confirmed in a larger series, HER2 overexpression may play a role not only in the development and progression of a subset of thyroid carcinomas, but also in their prognostic and therapeutic stratification. 

## 4. Materials and Methods

### 4.1. Sample Collection

Ninety sporadic differentiated thyroid tumors (DTCs) (45 papillary (PTCs) and 45 follicular (FTCs) thyroid cancers) with available formalin-fixed paraffin-embedded tissue blocks were selected from the files of the Department of Pathology of our University Hospital. The surgical samples were from 90 patients (73 F and 17 M, aged 28–76 years) who were diagnosed and followed up at the Endocrine Unit of our University Hospital over the last twenty years. The clinical records of the 90 patients were reviewed.

Histological classification was performed by two pathologists with experience in thyroid pathology (Giovanni Tuccari, Antonio Ieni and Giovanni Branca), according to the World Health Organization guidelines [34]. Institutional review board approval was obtained.

### 4.2. Immunohistochemistry

For each case, 5-µm-thick sections from representative tissue blocks of the tumor were obtained. Immunohistochemistry (IHC) was performed twice on each specimen using, firstly, the monoclonal antibody against HER2-pY-1248 (Phosphorylation site specific) (clone PN2A, Dako; w.d. 1:100) and, successively, the Hercep Test (Dako, Glostrup, Denmark), with an automated procedure (DAKO Autostainer Link48), according to the manufacturer’s instructions. An antigen retrieval pre-treatment was performed in 3 cycles in a 0.01 M citrate buffer, pH 6.0, in a microwave oven at 750 W. Staining intensity, the percentage of positive cells, and cellular localization were evaluated both in the tumor and in the adjacent non-neoplastic thyroid tissue. 

Since there are no established criteria for thyroid cancer, we adapted the current breast criteria for scoring HER2 in our DTC. Staining intensity and cellular localization in the tumor were evaluated and scored according to the updated ASCO-CAP scoring system for breast cancer [10]. Accordingly, the degree of HER2 staining was scored from 0 to 3+. HER2 positivity was defined as 3+ when strong membranous staining was noted in at least 10% of cells, 2+ when weak to moderate complete membranous staining was evident in 10% of tumor cells, 1+ when a faint or weak and incomplete membrane staining was observed, and 0 when no staining was observed or when staining was present in less than 10% of neoplastic cells. 

Immunohistochemical evaluations were carried out twice and blindly by two pathologists (Giovanni Tuccari, Antonio Ieni and Giovanni Branca). In the case of disagreement, cases were jointly discussed using a double-headed microscope until agreement was reached.

### 4.3. Fluorescence In Situ Hybridization

In cases showing 2+ immunostaining, as determined by IHC with the Hercept test, fluorescence in situ hybridization (FISH) analysis was performed using a HER2 FISH PharmDx™ kit (Dako, Glostrup, Denmark), according to the manufacturer’s instructions, to detect amplification of the *HER2* gene, as for breast cancer [35]. Gene amplification was recorded when the ratio HER2/centromeric probe for chromosome 17 (CEP17) signal was ≥2.0. 

**Figure 4:**
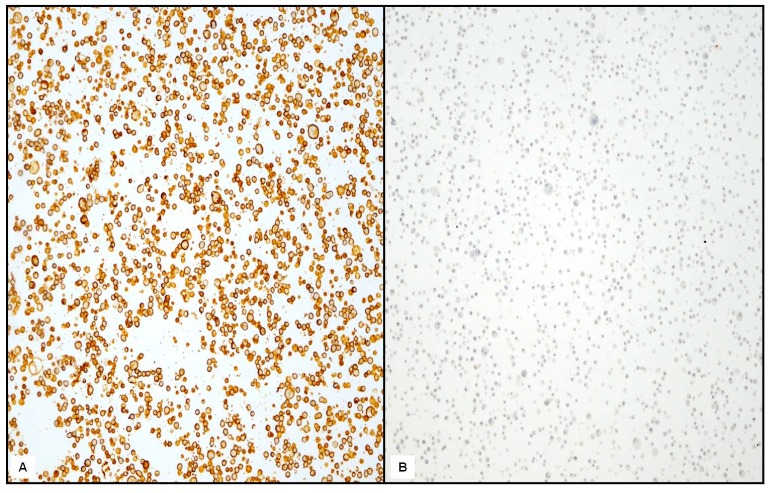
Breast carcinoma tissue section as positive control (**A**, 200×) and negative control (**B**, 200×).

Specimens of breast carcinoma were used as appropriate positive controls for both IHC and FISH analysis (Figure 4A). Negative controls were obtained either by omitting the primary antiserum or by replacing the primary antiserum with normal mouse serum, in a parallel section of the same cases (Figure 4B).

### 4.4. Statistical Analysis

Once tested for normal distribution and variance, data (mean ± standard deviation) were analyzed by the two-tailed Student’s *t*-test, a chi-square test with Yates’ correction for continuity, and linear regression analysis. The level of statistical significance was always set at *p* < 0.05.

## Figures and Tables

**Table 1:** Clinical and pathological features of the 90 differentiated thyroid cancer (DTC) patients at the time of surgery.

Variables	PTC Cases (*n* = 45)	FTC Cases (*n* = 45)
Age (years, mean ± SD)	50.6 ± 12.3	52.7 ± 13.2
Sex		
Male	11	6
Female	34	39
M:F	1:3	1:6.5
Histological features	Classic variant, *n* = 16	Minimally invasive, *n* = 34
	Follicular variant, *n* = 21	
	Hürtle cell variant, *n* = 4	Widely invasive, *n* = 11
	Sclerosing variant, *n* = 4	
Primary Tumour pT [29]		
T1	34 (13 T1a and 21 T1b)	21 (9 T1a and 12 T1b)
T2	10	13
T3	1	11
T4	/	/
Node metastasis (NX/N0/N1) [29]		
pNX	12	20
pN0	23	25
pN1	10 (N1a)	/

The symbol/means no case.

**Table 2:** Human epidermal growth factor receptor 2 (HER2) expression in thyroid cancer tissue *.

HER2 Status	FTC (*n* = 45)	PTC (*n* = 45)	*p*
IHC Negative/low (0, 1+)	12 (26.6%)	30 (66.6%)	0.020
Equivocal (2+)	15	7	
Positive (3+)	18	8	
IHC/FISH			
Positive cases			
(3+ and amplified)	20 (44.4%)	8 (17.7%)	0.046

* HER2 status has been assessed by using immunohistochemistry (IHC) to detect protein expression. Fluorescence in situ hybridization (FISH) was also performed in cases that tested 2+ at IHC, as specified in the Materials and Methods section to detect gene amplification.
